# Antimicrobial and Anti-Infective Activity of Natural Products: Unveiling Mechanisms, Synergies, and Translational Applications

**DOI:** 10.3390/antibiotics14111163

**Published:** 2025-11-17

**Authors:** Elizabeth S. Fernandes, Carmem D. L. Campos, José L. Pereira-Filho, Cinara R. A. V. Monteiro, Valério Monteiro-Neto

**Affiliations:** 1 Department of Animal and Veterinary Sciences-ANIVET Ruminant Nutrition (RUN), Aarhus University, 8830 Tjele, Denmark; elizabeth.fernandes@anivet.au.dk; 2 Programa de Pós-Graduação em Ciências da Saúde, Centro de Ciências Biológicas e da Saúde, Universidade Federal do Maranhão, São Luís 65080-040, MA, Brazil; carmem.campos@discente.ufma.br (C.D.L.C.); jlp.filho@discente.ufma.br (J.L.P.-F.); cinara.monteiro@discente.ufma.br (C.R.A.V.M.)

## 1. Introduction

Antimicrobial resistance (AMR) is an urgent global challenge as microbial pathogens are increasingly developing resistance to traditional antibiotics. Consequently, infections and deaths caused by these microorganisms have increased, but the development of new antimicrobial agents remains slow and limited [1,2,3]. This significantly affects the success of available treatments and the capacity of medical systems to manage these infections effectively.

The coronavirus disease (COVID-19) pandemic has exacerbated this issue, significantly contributing to concerns regarding the rise in transmission and emergence of AMR [4,5]. In response to this escalating threat, the World Health Organization has updated the list of bacterial [2] and fungal [1] priority programmes to enhance efforts and investments aimed at preventing the development of AMR, implementing public health policies, and conducting laboratory analyses and research to develop new drugs.

In this context, natural products and derived compounds, such as those sourced from plants, animals, and microorganisms, have historically been crucial to the discovery and development of new antimicrobial agents, with many drugs originating from these sources [6]. Building on the success of our previous edition, we present herein, the second special edition titled “Antimicrobial and Anti-Infective Activity of Natural Products.” This new collection of articles seeks to highlight recent research on the development of new antimicrobial compounds from natural products, mechanisms of action of pure compounds, in silico evidence of antimicrobial activity, synergistic associations with antibiotics, antimicrobial effects of probiotics and other microorganisms (and their products), and compounds with anti-virulence activity or the ability to neutralize microbial resistance. Prospects for the clinical application of these agents have also been explored, underscoring their therapeutic potential against the current challenges of AMR.

In total, 12 research and 2 review articles were published in this special section, and their conclusions are summarized below.

## 2. Extracts and Compounds of Natural Origin: From Antimicrobial and Immunomodulatory Actions to Possible Activities in Association with Other Medicines

Data discussed in articles on this Special Issue demonstrate the ability of plant extracts and compounds of natural origin to inhibit pathogens and modulate the immune system. They also investigated their anti-virulence activity and explored the use of these compounds in combination with other drugs.

Three papers reported the antifungal actions of natural extracts and isolated compounds against *Candida* spp. The study conducted by Costa et al. [7] reported the anti-*Candida* effects of the hydroethanolic extract of *Vismia guianensis* (EHVG) in *Tenebrio molitor* larvae and Swiss mice. The extract demonstrated antifungal activity and the ability to interfere with essential virulence factors of *Candida* spp., increasing the life expectancy of *T. molitor* larvae and infected mice. The authors also found that the extract exhibited low toxicity in vivo and immunomodulatory activity, providing crucial evidence for future clinical applications of the extract.

In another study by Mendes et al. [8], ellagic acid (EA), a natural dilactone of hexahydroxydiphenic acid, exhibited anti-*Candida* activity and potentiated the inhibitory action of fluconazole in resistant and sensitive strains of *C. albicans*. In parallel, the compound was able to modulate *Candida* morphological transition and biofilm formation, while presenting low toxicity to AS. Based on these results, the authors discussed the promising use of EA as a natural adjuvant to enhance the efficacy of fluconazole against *Candida*-resistant strains.

Sumlu et al. [9] integrated laboratory experiments and computer simulations to investigate the antifungal potential of artemisinin against *Candida* strains. This compound was found to suppress the expression of genes related to fungal adhesion and hyphal formation, both of which are involved in biofilm formation. These findings were supported by molecular docking and field-emission scanning electron microscopy analyses, demonstrating the activity of artemisinin in biofilm-associated structures. This study indicated that this compound may contribute to a new, effective antifungal therapy.

Two other studies explored the immunomodulatory and antimicrobial effects of natural compounds. Rodrigues et al. [10] evaluated the effects of punicalagin in combination with meropenem in a murine model of sublethal sepsis. The authors observed that this association promoted a significant increase in IL-10 levels and reduced bacterial translocation and organ damage. The results of this study highlight the potential of punicalagin as an adjuvant agent capable of increasing antibiotic efficacy and the immune response during sepsis treatment.

In another study, Kim and Kim [11] demonstrated the effect of quercetin, which, despite having documented antimicrobial activity, induces *Staphylococcus aureus* dormancy by triggering ATP depletion, thus increasing bacterial persistence. The authors pointed out that these findings highlight the need for therapeutic strategies that harness the antimicrobial benefits of quercetin without promoting bacterial persistence.

## 3. Essential Oils (EO): From the Laboratory to Therapeutic Application

The therapeutic use of essential oils as antimicrobials has been the subject of several studies in this Special Issue, highlighting their promising antimicrobial properties in various experimental and clinical contexts.

Schneider et al. [12] evaluated the efficacy of several base ointments containing West Indian lemongrass (*Cymbopogon citratus*) essential oil (LEO) in the treatment of pitted keratolysis (PK) in vivo, caused by pathogens such as *Kytococcus sedentarius*, *Dermatophilus congolensis*, and *Bacillus thuringiensis*. Among the tested ointments, the formulation with suspended LEO based on hydrogelum methylcellulose demonstrated the best antibacterial activity, in addition to prolonged stability and efficacy for up to two years. The in vivo therapeutic test of the ointment was performed in a patient with a chronic history of PK and revealed clinical improvement from day 1 of treatment, with disappearance of symptoms after 3 days and absence of bacterial load after the 4th day of treatment. These results indicate the antimicrobial potential of oil as an effective, practical, and affordable topical formulation.

In another study, Morocho et al. [13] evaluated essential oils of leaves and fruits of *Zanthoxylum mantaro* (J.F.Macbr.) J.F.Macbr. The authors determined the chemical composition of the oils, finding 23 compounds in the essential oil from the fruits and 47 compounds in the essential oil from the leaves. The major constituents of the fruit essential oil were α-thujone, β-thujone, sabinene, and terpinen-4-ol, whereas the main compounds in the leaf essential oil were germacrene D, nerolidol (E), and pentadecanal. Fruit EO exhibited antifungal activity against *Aspergillus niger*, whereas both fruit and leaf EO showed moderate antioxidant activity. These findings support future investigations into the therapeutic potential of EOs from *Z. mantaro* leaves and fruits as antifungal agents.

The work conducted by Chakraborty et al. [14] investigated the antimicrobial potential of cineole and 26 homeopathic medicines against planktonic cells and biofilms of drug-resistant clinical strains of *P. aeruginosa*. The authors highlighted the antibiofilm activity of the homeopathic medicine *Hypericum perforatum* (HyPer). Although cineole alone did not have the same effect when used in combination, it potentiated the antimicrobial and antibiofilm activities of HyPer. This was confirmed by molecular docking analysis. The authors suggested that these actions may be related to alterations in membrane potential, increased permeability of the outer membrane, and the release of bacterial DNA. The results indicate a potential therapeutic application of HyPer combined with cineole to combat *P. aeruginosa* resistance to drugs.

## 4. Antimicrobial Metabolite-Producing Microorganisms: From Identification to Application

The antimicrobial potential of the microorganisms and their metabolites was investigated in three studies. The analyses were carried out using molecular biology, in silico, and in vitro tools to provide interesting evidence of the promising antimicrobial potential of these microorganisms.

The integration of metabolomics and genomics was performed by Molina et al. [15] on the lineage *Lactiplantibacillus plantarum* UTNGt2. The combination of LC-MS/MS capillary metabolomics with the prediction of ribosome synthesis and post-translationally modified peptide (RiPP) gene clusters, combined with genomic annotation, has allowed the identification of several functional metabolites, such as organic acids with antimicrobial activity and biosynthetic clusters of peptides known for their antimicrobial and antitumor activities. Using this approach, the authors highlighted the promising potential of *L. plantarum* UTNGt2 and its metabolites for the development of antimicrobial agents, functional foods, and other biotechnological innovations.

Ma et al. [16] presented in their study the identification of a new species of *Bacillus*, *B. maqinnsis* sp. nov. Bos-x6-28 was isolated from the feces of free-grazing yaks in Maqin County, Qinghai Province, China. The use of the antiSMASH online tool revealed the biosynthetic potential of this strain to produce several bioactive compounds, including derivatives of fengycin and lichenysin, which are known to have antimicrobial, antitumor, and neuroprotective activities. These bacterial strains exhibited promising antitumor and antimicrobial activities. These results highlight the potential of this bacterial strain to synthesize bioactive natural products with antibiotic and anticancer activities.

In a study conducted by Albisoru et al. [17], the antimicrobial effects of polyketides produced by three species of *Monascus* were evaluated using in silico and in vitro approaches. Docking analyses revealed a high affinity of these polyketides for dihydrofolate reductase present in *S. aureus*, *E. coli*, and *C. albicans*. This was supported by in silico predictions, indicating that these compounds could be active against *S. aureus* (including MRSA), *E. coli*, and *C. albicans*. The authors pointed out that all the investigated compounds have the potential to cross the blood–brain barrier and recommended their potential application in topical antimicrobial formulations.

Palacios-Rodriguez et al. [18] evaluated the antimicrobial activity of *Bacillus* strains against *Escherichia coli* ATCC 25922. Among the isolates, *B. amyloliquefaciens* BS4 showed the highest antimicrobial activity. The authors also determined the physicochemical properties of the BS4 culture medium composition and found that it significantly improved the production of antimicrobial metabolites, resulting in larger inhibition zones against *E. coli* ATCC 25922. The results also showed antioxidant properties with no hemolytic toxicity, reinforcing the potential of these metabolites for future therapeutic applications.

## 5. Comprehensive Reviews on Natural Compounds with Antimicrobial Potential

This Special Issue also features two comprehensive review articles aimed at discussing the antimicrobial potential of natural extracts and compounds and their therapeutic applications and mechanisms of action.

The review conducted by Guerra et al. [19] presented an overview of the antifungal activities of extracts and compounds isolated from Anacardiaceae species, focusing on their mechanisms of action and potential therapeutic applications, particularly against *Candida* infection. Twenty-one Anacardiaceae species are discussed. The isolated compounds have the potential to treat fungal infections, while acting as anti-inflammatory, antioxidant, and apoptotic molecules.

Pereira-Filho et al. [19] reviewed and discussed the antimicrobial capacity of silybin, a flavonolignan extracted from *Silybum marianum* seeds (milk thistle). The authors highlighted the activity of silybin against a wide range of pathogens involved in mucosal, skin, gastrointestinal, and respiratory infections, indicating its antibacterial (especially Gram-positive), antifungal, antiviral, and antiparasitic activities. In addition to its antimicrobial effects, its potential for use in combination with other antimicrobial drugs is noteworthy. However, the authors pointed out that low compound bioavailability represents a significant challenge, requiring the development of formulations to overcome this limitation and enable the future clinical applications of silybin.

## 6. Final Considerations

In summary, this Special Issue of antibiotics assembles a pertinent collection of studies elucidating the broad antimicrobial potential of natural products, including plant extracts, purified compounds, essential oils, and microbial metabolites.

The compiled studies demonstrate that it has become increasingly necessary to pursue novel strategies to counteract antimicrobial resistance, and a multifaceted approach is required, ranging from in vivo to in silico modelling, to advance knowledge on the bioactive activity of compounds and their ability to either surpass or aid the use of antimicrobials and/or immunomodulators in treating infectious diseases.
